# Computer-Aided Surgical Simulation for Yaw Control of the Mandibular Condyle and Its Actual Application to Orthognathic Surgery: A One-Year Follow-Up Study

**DOI:** 10.3390/ijerph15112380

**Published:** 2018-10-27

**Authors:** Ju-Won Kim, Jong-Cheol Kim, Kyeong-Jun Cheon, Seoung-Won Cho, Young-Hee Kim, Byoung-Eun Yang

**Affiliations:** 1 Department of Oral and Maxillofacial Surgery, Hallym University College of Medicine, Anyang 14066, Korea; kjw9199@hanmail.net (J.-W.K.); ddskjc@hanmail.net (J.-C.K.); shgusbabo@naver.com (K.-J.C.); kotneicho@gmail.com (S.-W.C.); 2 Graduate School of Clinical Dentistry, Hallym University, Chuncheon 24252, Korea; kcallas2@gmail.com; 3 Institute of Clinical Dentistry, Hallym University, Chuncheon 24252, Korea; 4 Mir Dental Hospital, Daegu 41940, Korea; 5 Department of Image Science in Dentistry, Hallym University College of Medicine, Anyang 14066, Korea

**Keywords:** craniofacial, computer-aided simulation, virtual surgery, orthognathic, cone-beam computed tomography

## Abstract

*Background*: Favourable occlusal interdigitation and an optimized position of the mandibular condyle after surgery are essential for obtaining favourable results. The position of the condyle is determined during the operation. However, it is difficult to maintain the condyle’s original position post-surgery despite the efforts of the surgeons. Indeed, a degree of rotation of the condyle is unavoidable, since it is difficult to verify whether the condyle is positioned correctly during surgery. *Purpose*: To maximize contact between the bone segments, the condyle was rotated around the vertical axis using surgical simulations. We examined changes to the condyle-fossa relationship after comparing virtual surgery to actual surgery. *Methods*: From 2015 to 2017, 20 patients were diagnosed with skeletal malocclusion and participated in computer-aided surgical simulation before undergoing orthognathic surgery. In the simulation, the mandibular condyles were rotated around the vertical axis, and the proximal segments were fixed to the distal segments using a customized miniplate and positioning device during actual surgery. This study investigated the relationship between the condyle and fossa using cone-beam computed tomography for several different time periods (preoperative (T0), virtual surgery (Tv), postoperative three days (T1) and one year (T2)). *Results*: The coronal and sagittal view exhibited significant differences in the mean values between T1and T0, Tv, and T2 for all joint spaces. As a result of the distance, the mean value of T2 in both the superior joint space (JS) and the lateral JS was significantly higher than that of Tv. In contrast, the mean value of Tv in the medial JS was significantly higher than that of T2. Moreover, the mean value of T2 on the axial plane was significantly larger than the values of Tv and T1. The mean value of T0 was also significantly larger than those of Tv and T1, and the mean value of Tv was larger than that of T1. Although the condyle was rotated, it exhibited a tendency to return to its preoperative position. There was no statistically significant difference in functional evaluation between T0 and T2. *Conclusion*: Our method of using yaw control for the condyle during virtual surgery and transferring this technique to the actual surgery can improve the conventional surgical technique by positioning the proximal segment in a pre-planned position, thus achieving optimal results.

## 1. Introduction

The goal of successful orthognathic surgery (OGS) is to correct malposition of the jaw bone, resolve related malocclusions, achieve aesthetic correction of the facial appearance, and to treat problems related to the upper respiratory tract [1,2]. Class III malocclusions comprise approximately 48% of the total number of malocclusions in the Republic of Korea. The majority of patients with class III malocclusion cannot be treated solely by orthodontic treatment and are instead treated through OGS [3]. Particularly for patients with facial asymmetry, the mandibular angle and ramus on both sides exhibit dissimilarities, causing mandible displacement and deviation [4]. After the first report of sagittal split ramus osteotomy (SSO) by Obwegeser [5], SSO has been used to treat various mandibular deformities, including mandibular prognathism. It has the advantage of rapid healing after surgery because of the large contact area between the bone segments. However, even with the identical method of osteotomy, the postoperative results may vary depending on several factors, including the surgical proficiency of the surgeon, the vector of the distal segment (DS, the tooth-bearing segment) during surgery, the bone fixation method used, altered soft tissue tension after surgery, the stability of the postoperative occlusion, the postoperative position of the mandibular condyle (hereinafter referred to as just the “condyle”), and the amount of contact and interference between the proximal segment (PS, mandibular condyle bearing segment) and the DS [6,7,8]. Among these factors, only the position of the condyle is determined by a surgeon during surgery. The position of the PS can be influenced by the mandibular plane angle and the anatomical conformation of the mandible, such as a U or V shape [9]. Discrepancies in the amount of bilateral displacement and the ramus angle degree between the two sides also contribute to the interference, or gap, between the PS and DS in patients with facial asymmetry [10,11,12]. In other words, changes in the position of the condyle are inevitable due to the use of screw or miniplates to fix the bony segments in place, thus increasing the risk of postoperative complications, such as an early relapse or temporomandibular disorder (TMD).

One study reported that maintenance of the preoperative anatomical positions of the condyles and adjacent proximal mandibular ramus segment after SSO is important [13]. Therefore, surgeons strive to maintain the pre-surgical position of the condyle as much as possible. However, a degree of displacement is unavoidable despite attempts to maintain the original preoperative position [14,15]. Additionally, such attempts can cause invasive problems. In one study, the transcutaneous pins were inserted into the necks of the right and left condylar process of the mandible and the supraorbital ridge to track the position of the condyle [16]. Other proposed approaches to minimize change in condylar position include the removal of the interference in the lingual surface of the PS; bone grafting to maintain the space between the PS and DS; and use of the short lingual technique, distal cutting technique, and vertical osteotomy in the third molar area within the DS [14,17,18,19,20,21]. Moreover, the position of the condyle during surgery can be influenced by the experience of the surgeon and a variety of condylar positioning devices [22,23].

Traditional dental-care techniques have worked excellently for decades. However, if a doctor wants a simpler, faster, more accurate, and more efficient workflow, then digital dentistry shows great potential. Indeed, introducing digital technology is essential, as traditional methods of performing various simulations for OGS are limited. The introduction of a whole range of digital data acquisition devices (cone-beam computed tomography (CBCT) [24], intraoral [25] and desktop scanner, and face scanner [26]), planning software (computer-assisted design and computer-assisted manufacturing software, software for guided implant surgery) [27], new aesthetic materials [28], and powerful fabrication machines (milling machines, 3D printers) is radically changing the dental profession [29]. The introduction of these technologies allows the movement of the jaws to be measured during virtual surgery and then accurately reflected during actual surgery.

In this study, a three-dimensional (3D) computer-aided surgical simulation (CASS) was used to minimize the interference or gap between the PS and DS during surgery. The surgical simulation was performed after a hybrid skull-dental cast model (Virtual Face) was created with (CBCT) and a model scan. During OGS, the segmented facial skeletons were rotated in three directions (pitch, roll, and yaw) based on the three axes (horizontal, axial, and vertical axis) and subsequently moved back and forth, left and right, and up and down. Since the facial bone is intricately moved during OGS, three aeronautical rotational descriptors (pitch, roll, and yaw) were used to supplement the planar terms (anteroposterior, transverse, and vertical) to describe the orientation of the line of occlusion and the esthetic line of the dentition [30]. In most patients with dentofacial deformity, yawing of the condyle occurs when the PS is rotated to be fixed to the DS at the appropriate position. Condyle yawing in the mandible is inevitable, and its degree is unpredictable. Therefore, we performed only the yaw rotation to avoid pitch and roll movement of the proximal segment during virtual surgery. Yaw rotation refers to rotational movements around the vertical axis [31]. The maximum amount of condyle yaw rotation was set to a smaller amount than that in previous studies [18,19,32]. To apply the results of virtual surgery to actual surgery, miniplates and a ramus positioning device were designed and then manufactured using a computer numerical control (CNC) milling machine and a 3D printer. This study aimed to verify the physiological adaptation of the rotated condyle, given that a positional change in the condyle is inevitable during OGS. An appraisal of the function in this position and its effect on the temporomandibular joint (TMJ) was also conducted.

## 2. Materials and Methods

### 2.1. Patients

The inclusion criteria consisted of the following: (1) patients who had previously undergone OGS between January 2015 and April 2017, (2) those with pre-surgical orthodontics, and (3) those for whom at least one of the jaws was operated. The exclusion criteria included (1) patients with cleft palate or craniofacial syndromes, (2) patients for whom a proximal segment positioning device (PSPD) was not used during surgery, and (3) patients presenting TMJ disease before the surgery. A single surgeon operated on all of the patients. All of the subjects’ medical records and radiographs were analyzed. This study was conducted in accordance with the World Medical Association Declaration of Helsinki on medical research ethics. Study approval was granted by the Hallym University Sacred Heart Hospital’s institutional review board (IRB No. 2018-I010), and informed consent was obtained from every patient.

### 2.2. Virtual Surgery and Actual Surgery

**Figure 1:**
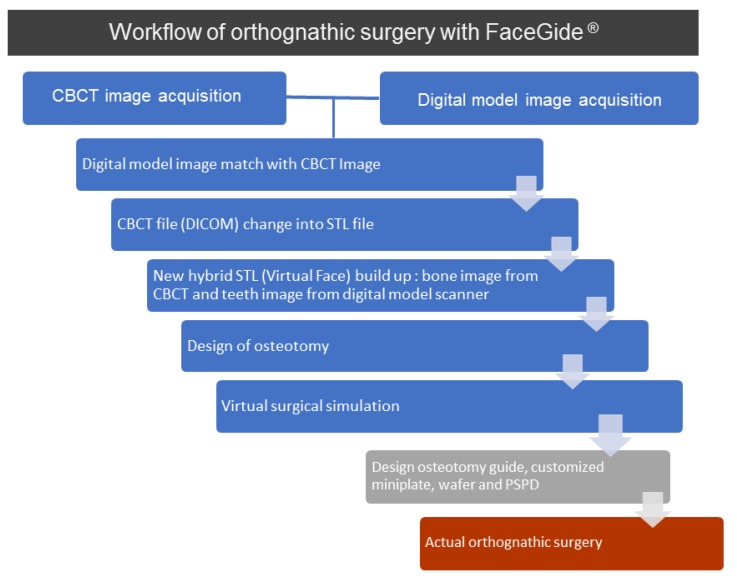
Our protocol for orthognathic surgery with FaceGide^®^ was as follows: a clinical examination and 3D data acquisition (a cone-beam computed tomogrphy (CBCT) image and an image of the patient’s dentition using a 3D model scanner) were performed, and a composite 3D virtual model (Virtual Face) was built. We quantified the deformity via 3D anthropometric analysis, and surgical simulation, including osteotomy and repositioning of the bone segments, was then performed. The osteotomy guides, customized fixation plates, surgical wafers, and proximal segment positioning device (PSPD) were designed and manufactured. A virtual surgical plan and the associated materials were delivered to the operating room.

Digital imaging and communication in medicine (DICOM) files were obtained from the CBCT results (Alphard 3030; Asahi Roentgen Inc., Kyoto, Japan). The DICOM files were converted to standard tessellation language (STL) files. All dental models were scanned using a desktop model scanner (Freedom HD, Dof Inc., Seoul, Korea), and the images were edited after the CBCT and dental model STL files were merged. Since the STL of the dental cast is more accurate than the CBCT image, it is necessary to edit the merged image. The edited images were converted back into an STL file and transformed into a 3D image using the FaceGide^®^ (Megagen Co. Ltd., Daegu, Korea) program for the virtual surgery. Figure 1illustrates a simple workflow of OGS performed using the FaceGide^®^ program, which is one of the CASS programs.

**Figure 2:**
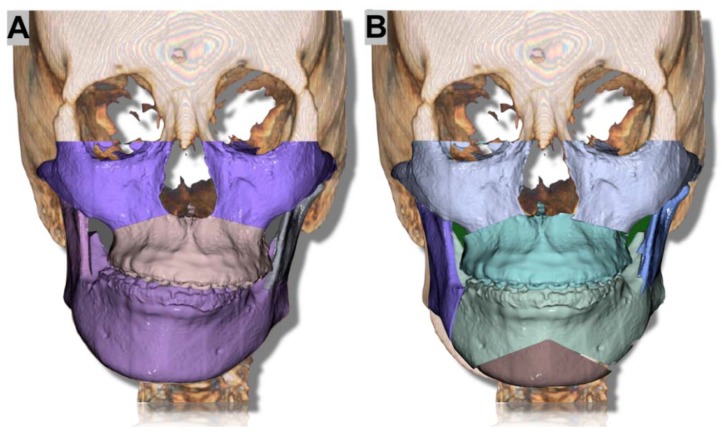
A 24-year-old patient underwent virtual simulation surgery for the improvement of facial asymmetry. (**A**) A split bone without any positioning and (**B**) the positioned segments.

Bilateral SSO was performed, and the interference and gap between the segments following the mandibular movement were measured in the virtual surgery. The virtual surgery was performed using FaceGide^®^ and Geomagic^®^ Touch™ X (3D Systems, Rock Hill, SC, USA) (Figure 2).

**Figure 3:**
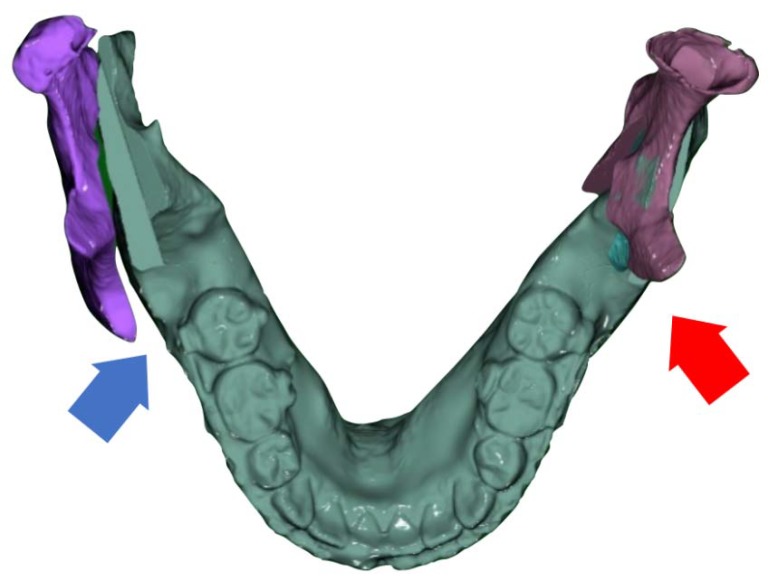
A gap (blue arrow) and interference (red arrow) can be observed between the proximal segment (PS) and distal segment (DS) after DS movement from virtual surgery.

Regarding the bimaxillary operation, a conventional LeFort I osteotomy for the maxilla was performed. The first step in the virtual surgery of the mandible was to confirm the width of the gap between the two segments after relocating the DS into a class I occlusion without moving the PS (Figure 3).

**Figure 4:**
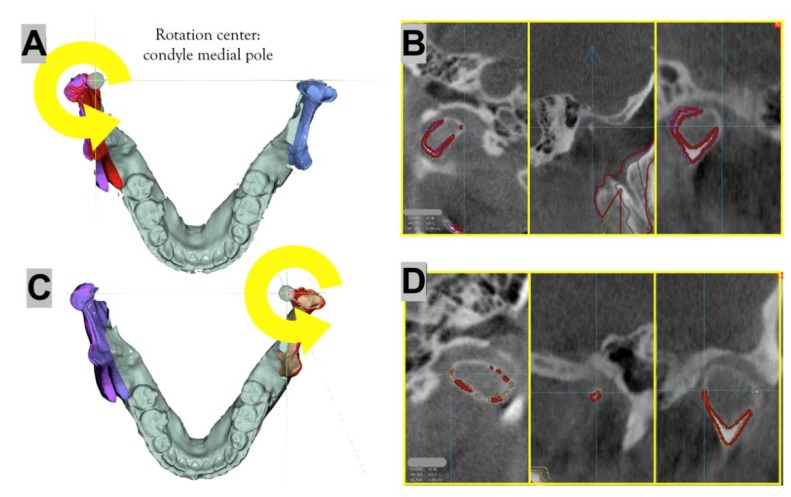
During the virtual surgery, the condylar head is rotated on the axial plane with respect to the medial pole of the condyle so that the mandibular fossa-condyle relationship is not severely impaired and there is minimal interference or space between the PS and DS. (**A**) Simulation surgery in the right condyle: the original condition (purple color) and medial rotation (red color) with the medial pole as the center are shown. (**B**) The red line shows the condyle outline after the virtual surgery was performed on the right condyle. (**C**) Simulation surgery in the left condyle: the original condition (yellow color) and lateral rotation (red color) with the medial pole as the center are shown. (**D**) The red line shows the condyle outline after the virtual surgery was performed on the left condyle.

Next, both mandibular condyles were rotated to ensure that maximum contact was secured between the segments. Any gap or interference was to be minimized in this procedure. After both proximal segments were rotated, the virtual surgery image of the condyle was superimposed onto the pre-surgical CBCT image to verify that the minimum amount of movement was achieved (Figure 4).

**Figure 5:**
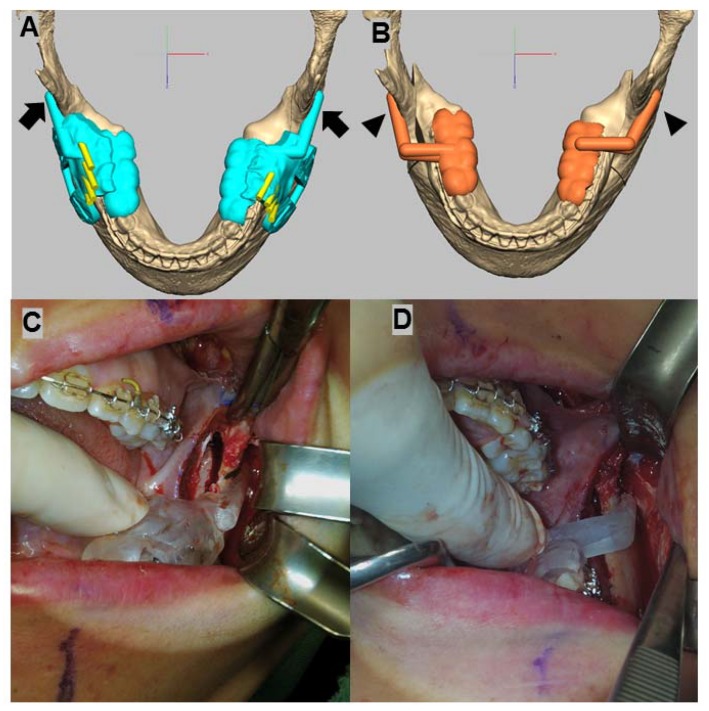
A ramus osteotomy guide (**A**) and a PSPD (**B**) designed for use with the virtual surgery of a 21-year-old patient are shown; a photograph shows the actual application of the osteotomy guide (**C**) and a PSPD (**D**). The PSPD arm (black arrowhead) is placed on the buccal surface of the ramus during the real surgery and should be placed in the same position as the arm (black arrow) of the ramus osteotomy guide.

Once the amount of yaw control of the PS was determined, two surgical guides were designed using Geomagic^®^ Freeform^®^ Plus (3D Systems, Rock Hill, SC, USA) and then printed using a D30 3D printer (Rapidshape GmbH, Heimsheim, Germany). One of the two guides was a ramus osteotomy guide (Figure 5A), designed based on a 3D image without moving any of the segments; the other was a PSPD (Figure 5B), designed based on a 3D image in which the bone segments (the PS and DS) had been moved.

**Figure 6:**
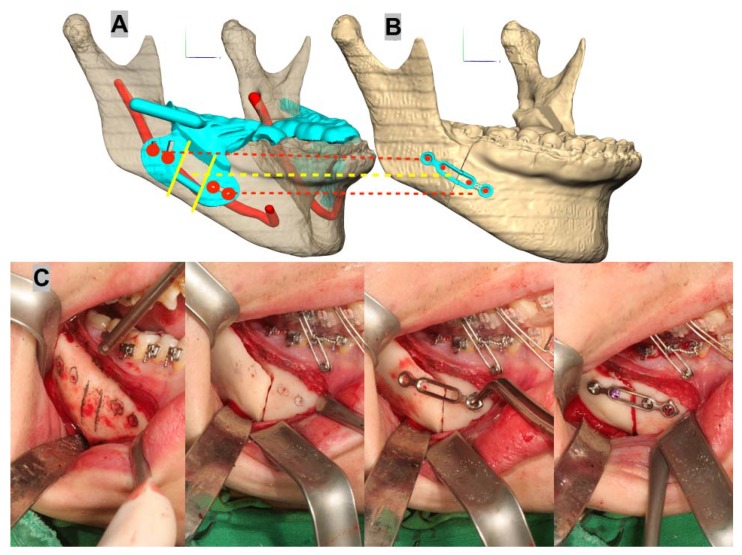
Virtual surgery images of the osteotomy guide (**A**) and customized miniplate (**B**) applied during mandibular surgery. Bony holes for insertion of the screw through the guide before the osteotomy and customized miniplate applied to preformed holes with PS and DS movement after the split of the ramus (**C**) are shown.

Additionally, the miniplates were customized to the patient, and designed and produced by CNC machines (ARDEN, TPS Korea Ltd., Gwangju, Korea). The final position of the PS was determined through virtual surgery, and the distance between the condyle and mandibular fossa and the degree of yaw control for both condyles was measured in the same manner as that used to perform measurements on the CBCT images. After performing the SSO during the actual surgery using an osteotomy guide (Figure 5C), the position of the PS that was set in the virtual surgery was reproduced using the PSPD (Figure 5D), and both segments were fixed with a customized miniplate (Figure 6).

### 2.3. Radiological Evaluation

**Figure 7:**
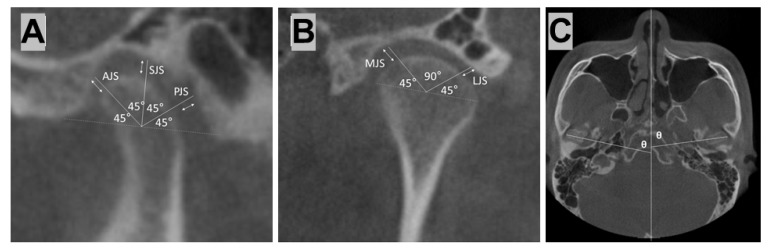
(**A**) In the sagittal view, the superior joint space (SJS), anterior joint space (AJS), and posterior joint space (PJS) distances were measured; (**B**) in the coronal view, the medial joint space (MJS) and lateral joint space (LJS) distances were measured; and (**C**) in the axial view, the condylar angle (θ) was drawn.

Cone beam computed tomography (CBCT) images of the subjects were obtained at preoperative (T0), postoperative three days (T1), and postoperative one year (T2). All of the images were obtained with the Frankfort horizontal plane parallel to the floor using a cephalostat. The TMJ was in the centric relation (CR) position using a CR bite at T0, and the CBCT settings were 80 kVp and 5 mA, with an exposure time of 17 s. The 3D image of the virtual OGS was set to Tv (T virtual). On-demand 3D software (CyberMed Co., Seoul, Korea) was utilized for the analysis of the condyle-fossa relationship, with reference points in the sagittal, coronal, and axial views. Two observers performed the measurements, and thedistance and angle were calculated as the average of the two observers’ measurements. Sagittal, coronal, and axial image slices were selected for each patient during the follow-up periods. For the sagittal image, measurements were taken as illustrated in Figure 7A. A reference line was placed tangentially to the lowest posterior and anterior extremities of the mandibular fossa. The midpoint between the front and back points of the condyle was designated the middle of the reference. The superior line was drawn from this point to the reference line at a 90° angle, and the anterior and posterior lines were drawn from this point to the reference line at a 45° angle. Three measurements were made from the image (anterior joint space (AJS), superior joint space (SJS), and posterior joint space (PJS): namely, the distances between the outermost point of the condyle and the closest points to the mandibular fossa that overlapped the superior line, anterior line, and posterior line). For the coronal image, measurements were taken as illustrated in Figure 7B. The most medial and lateral points of the condylar process were connected to produce a reference line. The midpoint of this line is referred to as the middle point of reference. The medial and lateral lines were subsequently drawn from this point at a 45° angle to the reference line. The medial joint space (MJS) and lateral joint space (LJS) measurements were obtained in the same manner as that used for the sagittal images. In the axial view, the angle (θ) between the line connecting the medial pole and lateral pole of the condyle, and perpendicular to the midpoint of the axial surface, was measured (Figure 7C).

### 2.4. Functional Evaluation

Patients were investigated for TMJ through clinical tests for maximum mouth opening (MMO) and lateral mandibular movement at T0 and T2. A ruler was placed at the most vertically oriented incisal edge of the maxillary central incisor to measure the MMO vertically to the labioincisal edge of the opposing mandibular incisor. The amount of vertical incisor overlap was added to each of these measurements to determine the actual amount of opening. The patient opened their mouth slightly and moved the mandible as far as possible to the right or left for lateral movement measurement. A ruler (millimeters) was used to measure the distance from the labioincisal embrasure between the maxillary central incisors and the labioincisal embrasure of the mandibular incisors.

### 2.5. Statistics

The distance between the condyle and mandibular fossa and the degree of the condylar rotation was analyzed at T0, Tv, T1, and T2. Repeated-measures ANOVA and the Bonferroni post-hoc test were conducted for statistical analysis. Moreover, MMO and lateral movement were statistically analysed using the Wilcoxon signed rank test. The data were subsequently analyzed using Statistical Package for the Social Sciences (SPSS, version 23.0, IBM Corporation, Armonk, NY, USA), in which *p*-values less than 0.05 were considered to indicate significance.

## 3. Results

**Figure 8:**
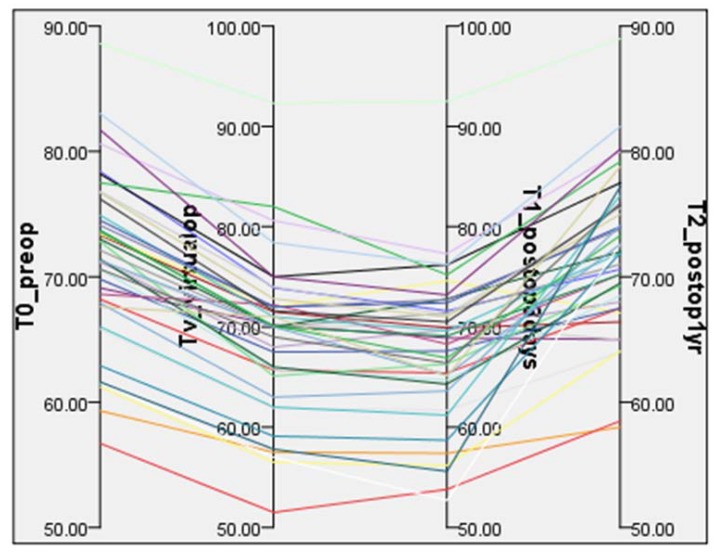
Changes in the condyle angle in the axial plane over time. T0 (preoperatively), Tv (virtual surgery), T1 (3 days postoperative), and T2 (1-year postoperative).

Twenty patients (nine males and 11 females) were eligible for inclusion and presented with skeletal malocclusion. The mean age of the patients was 24.55 years. Seven patients underwent a mandibular operation alone, and 13 patients further underwent a conventional LeFort I osteotomy. Bilateral SSO was conducted on all patients to position the DS of the mandible using Obwegeser’s technique [33] with the Epker modification [34]. Table 1 presents the mean and standard deviation of each measurement performed in our study. The statistical analysis revealed significant differences between the groups. A post-hoc analysis was conducted to examine these differences. The coronal and sagittal view also exhibited significant differences in the mean values between T1 and T0, Tv, and T2 for all joint spaces. As a result of the distance, the mean value of T2 in SJS and LJS is significantly higher than that of Tv. In contrast, the mean value of Tv in the MJS is significantly higher than that of T2. The mean value of T2 on the axial plane is significantly larger than the values of Tv and T1. The mean value of T0 is also significantly larger than those of Tv and T1, and the mean value of Tv is larger than that of T1 (Table 1, Figure 8). In the functional evaluation, the MMO before the operation ranged from 46 to 59 mm. One-year post-surgery, this range was 48 to 59 mm. The mean preoperative MMO was 53.40 ± 4.11 mm, and the maximum opening at one-year post-surgery was 53.45 ± 3.36 mm. The difference between the groups is not significant according to the Wilcoxon signed rank test (*p* = 0.887). The mean amount of lateral movement towards the right side was 12.60 ± 1.57 mm before the operation, while the mean value after one year was 12.60 ± 1.47 mm. However, the difference between the groups is not significant according to the Wilcoxon signed rank test (*p* = 0.882). The mean amount of lateral movement towards the left side was initially 12.30 ± 1.49 mm, while after one year, the mean value was 12.35 ± 1.14 mm. The difference between the preoperative and postoperative values is not significant (*p* = 0.398) (raw data provided in Supplementary Table S2). None of the 20 patients exhibited malocclusion, infection, or neural disorders one-year postsurgery.

## 4. Discussion

Many studies have demonstrated that the condyle position changes somewhat after OGS despite the efforts of clinicians to maintain the condyle’s original position [18,32,35,36]. In this study, we attempt to optimize the junctions between the bone segments by minimizing displacement of the condylar position on the yaw axis. A year after the operation, CBCT results indicate that the relationship between the condyle and fossa was similar to that before surgery. The functional test further revealed joint movements similar to those before the operation as well. Fixation of the PS and DS in an appropriate position is a prerequisite for minimizing the displacement of the condyle when locating the mandible. Condylar displacement has a strong influence on postoperative stability, relapse, TMD, joint sounds, and condylar resorption [15,18]. Meanwhile, condylar displacement can be attributed to several factors, including the presence of muscle or soft tissue around the mandible, the method used to relocate the condyle, interference between bony segments, and the fixation method used [37,38,39]. Furthermore, clockwise rotation of the PS in the pitch axis during surgery causes a stretched pterygomasseteric sling, thus leading to an anticlockwise rotation of the PS in the pitch axis postoperatively [40,41]. Therefore, it is reasonable to state that the postoperative position of the PS is crucial for postoperative stability, normal functioning of the TMJ, and the prevention of TMD. In the case of PS positioning without any form of external force to prevent condylar displacement, the gap between the PS and DS grows, reducing the contact between them. Conversely, a tight fit of the PS and DS results in lateral displacement of the condyle. A variety of surgical methods have been proposed to solve this problem [14,19,20,42]. However, many studies have claimed that, regardless of the method used, the displacement of the PS and condyle is practically inevitable [43]. Particularly in cases of facial asymmetry, medial or lateral rotation of the PS in the yaw axis is necessary when relocating the mandible into a symmetrical position. This yaw rotation of the PS, however, enlarges the degree of rotation of the bone segments when a gap or interference is formed between the segments, thus resulting in early relapse or TMD post-surgery. Ghang et al. have reported that the condyle rotated 1.78° medially on average during the surgery, while it further rotated 0.40° laterally during the stabilization stage [35]. Meanwhile, in a retrospective study on patients with facial asymmetry, Baek et al. have reported that the medial condylar rotation was as large as 7.90° on the side with the larger DS movement, and as large as 12.90° on the side with less DS movement. The mean medial condylar rotation for the sides were 2.80° and 2.32°, respectively [32]. Kim et al. have also reported that, compared with the pre-surgical position, the condyle was rotated 5.04° on the right side and 5.84° on the left side six months post-surgery [36]. These studies imply that the yaw rotation of the mandibular condyle is an inevitable outcome following DS positioning. When the miniplates are fixed on the bone, undesired stress can occur unless the miniplates are bent the optimal amount to prevent any excessive external force on the bony segments. In this study, the position of the PS was set within 5° of the maximum condylar rotation, considering both the minimum stress and maximum contact area during bone fixation through virtual surgery. Ellis et al. have expressed doubts regarding the optimized pre-surgical position of the condyle [44]. Thus, there is a need for rearrangement of the condyle-fossa relationship in patients with severe facial deformity. During virtual surgery, the lateral pole of the condyle was rotated around the medial pole to ensure that the condyle-fossa relationship remained consistent. To our knowledge, since no such attempt has been made before, little is known regarding the importance of designating the centre of rotation. A group of researchers have, however, reported that the anterior rotation of the lateral pole leads to a satisfactory result post-surgery [22]. Furthermore, Dawson has proposed that the medial pole is the only logical common rotation point that would permit a true rotation to occur on a fixed axis [45]. Based on these studies, we designated the medial pole of the condyle as the rotation point. In the present study, the measured values of the MJS and LJS were different. In the case of the MJS, the mean value at Tv was larger than that at T2. In contrast, in the LJS, the mean value at T2 was larger than that for Tv. This result is presumed to be related to the designation of the medial pole of the condyle as the centre of rotation. However, although Rotskoff et al. have reported significant improvement in the vertical and horizontal changes to the condylar position, no significant difference in the condylar rotation was determined when using a condylar positioning device [46]. Xi et al. have also revealed that postoperative flaring and torque were hardly related to skeletal relapse or condylar remodelling in 56 patients who underwent advanced SSO surgery [47]. These results support the notion that the yaw control of the condyle is possible. In our study, the mean difference in the axial plane between T0 and Tv was 2.61 ± 2.74 degrees, which is smaller than the yaw rotation of the condyle reported in previous studies. To reflect the virtual surgery in the real surgery, a PSPD was made using a 3D printer, and customized miniplates were constructed using a CNC milling machine and subsequently used in the actual surgery. When the PSPD and the customized miniplates are applied during surgery, they should be placed passively on the tooth part of the DS and the ramus buccal surface of the PS so that a strong force is not applied. In addition, a PSPD arm placed on the buccal surface of the ramus during surgery should be placed in the same position as that of the arm of the ramus osteotomy guide. This study is based on the fact that condylar movement following rotation of the PS is unpredictable, given that the maximum area of contact is ensured between the segments during the surgery and that, in many previous studies, the pre- and postsurgical condylar positions exhibit a difference. In general, the main purpose behind repositioning the condyle is to prevent condylar sagging [7,11,39,46]. In patients with asymmetry and those with a large movement of the DS, there is either interference between the PS and DS or an increase in the gap. In these cases, to increase the area of bone contact, the PS must be rotated depending on the experience of the surgeon during bone fixation. In this study, the rotational angle of the condyle and the optimal bone position were set during the virtual surgery based on the data presented in previous studies. Satisfactory results were obtained. However, the measured values at T1 were larger than those at other periods because of changes in the muscle function around the condyle and postoperative edema. The difference between the use of a wafer after the surgery and the final occlusion observed in one study [48] could also be a factor. Depending on the case, this factor can be eliminated by not using a wafer post-surgery. If a wafer is necessary, minimizing its thickness will help decrease the rotational error that can occur [48]. In this study, the condyle-fossa relationship one-year post-operation was similar to that at T0. Moreover, a new bone structural unit was reportedly made four months after bone surgery [49]. A year after the operation, the CBCT results indicate that the bone union was completed and that the condyle-fossa relationship remained almost unchanged. Furthermore, although the condyle rotation was performed based on the yaw axis, no difference in mouth opening motion was observed one-year post-operation compared to just before the operation. This result confirms the findings of previous studies, which suggest that condylar rotation within the range of a patient’s clinical adaptation does not cause TMD [22,50]. Our study was performed in patients without TMD, and the effect of yaw control on patients with craniofacial deformity with joint disease was not known at this time. Several researchers have argued that the centric relation position changes continuously or represents a range of positions, which limits our ability to identify such a position [51,52]. However, we also agree with this report, and it appears that a condyle position similar to that before surgery is clinically acceptable.

This study, however, has several limitations. First, the small sample size limits the ability to draw strong conclusions. One reason for the small sample size was the utilization of strict inclusion and exclusion criteria, which resulted in the exclusion of the majority of orthognathic surgeries completed in the department during the study period. Indeed, we excluded patients from the study who had TMD before surgery. Moreover, we did not use a PSPD in these patients, because we determined that yaw control of the condyle could have an adverse effect on the disease-bearing TMJ. Thus, further studies are needed in patients with TMD. Second, we did not designate a control group, because we were confident that this method was noninvasive and would help determine the optimal condyle position for each of the patients. Third, it is necessary to compare groups based on the amount of movement of the DS. Future studies should also use a larger patient population and compare the results of the group that underwent a bimaxillary operation and the group that underwent a single jaw operation. In addition, although we selected the medial pole as the centre of condylar rotation, future studies are needed to determine whether there is an ideal centre. To the best of our knowledge, this study is the first to perform a follow-up assessment of the condylar position after the yaw control of the PS with CBCT. In this study, the yaw control of the condyle was performed to optimize bone contact, and thus stable functional results were obtained postoperatively. Moreover, this study suggests the potential of patient-customized surgery via CASS.

## 5. Conclusions

In this study, the PS was located in the optimal position when the PS and DS were connected with the help of virtual surgery, and this positioning was reflected in the actual OGS. We have refined the conventional method of positioning the PS and overcome its limitation, namely, that the surgical results primarily rely on the experience of the surgeon. Furthermore, this study individually customized the condylar rotation, and we hope that these modifications will improve traditional methods.

## Figures and Tables

**Table 1:** Statistical analysis of the condylar positions in the sagittal, coronal, and axial views at T0, Tv, T1, and T2.

		T0		Tv		T1		T2					
		*n* = 40		*n* = 40		*n* = 40		*n* = 40					
		Average	SD	Average	SD	Average	SD	Average	SD	df	F	*p* ^(1)^	B ^(2)^
AJS	mm	1.92	0.7	1.89	0.7	2.6	0.79	2.07	0.63	1.72	25.44	0.00 *	T1 > T0, Tv, T2
SJS	mm	2.48	1.06	2.25	0.86	3.48	1.12	2.51	0.9	1.79	33.75	0.00 *	T1 > T0, Tv, T2
													T2 >Tv
PJS	mm	2.07	0.93	2.04	0.91	2.67	1.01	2.09	0.68	1.48	11.78	0.00 *	T1 > T0, Tv, T2
MJS	mm	2.28	1.09	2.46	0.91	3.37	1.07	2.25	0.7	2.27	39.35	0.00 *	T1 > T0, Tv, T2
													Tv > T2
LJS	mm	1.95	0.82	1.82	0.56	2.6	1.25	2.08	0.58	1.61	14.21	0.00 *	T1 > T0, Tv, T2
													T2 > Tv
angle	degree	71.64	6.66	69.03	7.65	67.93	7.44	71.98	6.22	1.49	15.82	0.00 *	T2 > Tv, T1
													T0 > Tv, T1
													Tv > T1

^(1)^ Statistical significances were tested by Repeated Measures ANOVA among groups (* *p* < 0.05). ^(2)^ Adjustment for multiple comparisons: Bonferroni.

## Supplementary Materials

All data generated or analyzed during this study are included in this published article (and its supplementary information files). The following are available online at http://www.mdpi.com/1660-4601/15/11/2380/s1, Table S1: Raw data for CBCT measurements, Table S2: Raw data for oral functional evaluation.
