# Supplementary material for: Computer-Aided Surgical Simulation for Yaw Control of the Mandibular Condyle and Its Actual Application to Orthognathic Surgery: A One-Year Follow-Up Study
Source: Int J Environ Res Public Health. 2018 Oct 27;15(11):2380. doi: 10.3390/ijerph15112380 (PMC6267607; doi:10.3390/ijerph15112380)
Supplement: Supplementary file 1 [file ijerph-15-02380-s001.pdf]

Supplementary Table S1 Raw data for CBCT measurements

	MJS								LJS							
	T0 Rt.	Tv Rt.	T1 Rt.	T3 Rt.	T0 Lt.	Tv Lt.	T1 Lt.	T3 Lt.	T0 Rt.	Tv Rt.	T1 Rt.	T3 Rt.	T0 Lt.	Tv Lt.	T1 Lt.	T3 Lt.
pt01	3.1	3.8	4.2	3.8	3.2	4	4.4	3.8	1.5	1.6	1.7	1.6	1.8	1.8	1	1.8
pt02	2.4	2.4	2.5	2.2	2.1	2.3	2.2	2	1.9	2	2.2	2.2	2.3	2.5	2.2	3.2
pt03	1.3	2.8	3	2	1.4	2.5	3.4	2	1.6	1.9	2.5	1.8	1.5	1.7	1.9	1.8
pt04	4.8	4.8	4.9	3.9	6.1	3.9	4.9	2.6	2.4	2.9	3.9	3.2	2.7	2.8	1.8	2.5
pt05	1.8	1.7	1.9	1.9	1.9	2	3.9	2.3	1.4	1	0.8	1.2	2.2	2.2	2.3	2.7
pt06	1.9	2	2	1.9	2	1.9	2.5	2	1	2.1	3.1	1.8	1.2	1.8	2	1.9
pt07	2.8	3.5	3.6	3.4	4.7	4.9	5.6	3.2	2.4	2.3	3.1	2.5	1.9	1.8	2.5	2
pt08	2.3	2.3	4.4	2.4	1.9	1.9	4.2	2	1.2	1.2	1.2	1.8	3.1	3.1	4	3
pt09	1.6	1.7	3.1	1.7	1.6	1.7	4.4	1.5	1.4	1.4	2.1	1.8	1.5	1.5	1.3	1.6
pt10	1.8	2.3	3.6	2.4	1.3	1.7	3.3	1.5	1.7	1.4	4.2	2.1	1.7	1.8	3.2	1.9
pt11	1.8	1.8	3.3	2.4	1.8	1.8	2.7	1.9	1.3	1.3	3.5	2	1.1	1.1	1.9	0.9
pt12	2.3	2.3	2.8	2.2	2.9	2.3	2.8	2.6	2	2	2.1	2.4	2.4	2.8	2.7	2.7
pt13	1.5	2.1	2.5	1.6	1.8	1.6	3.2	1.4	1.1	1	1.5	1.2	3	1.8	4.4	2.7
pt14	1.1	2.1	2.2	1.8	0.9	1.4	2.3	1.2	0.7	1	0.9	1	1	1.1	1.1	1.1
pt15	1.8	1.8	3	1.5	1.2	1.4	3.2	1.8	2.5	1.6	4.1	2.6	1.3	1.3	3	2.3
pt16	1.2	1.2	2.2	1.4	3.9	3.8	6.4	2.3	1	1	2.4	1.2	3.9	2.4	7.3	2.8
pt17	2.8	3.5	4	3.1	3.4	3.1	4.2	3.4	1.6	1.6	1.1	2	2.7	1.8	2.6	2.1
pt18	1.5	1.7	1.7	1.6	2.2	2.4	2.5	2.3	2	2.3	3.2	2.2	3.5	2.4	2.8	2.5

pt19	2.7	2.8	3.4	2.5	1.8	2.4	4.7	1.6	2.5	2.3	3.5	2.6	4.2	1.7	4	2.3
pt20	2.8	2.7	3.2	2.7	1.7	2.2	2.3	2	2.4	1.9	2.6	2.2	1.5	1.4	2.3	2

	AJS								SJS							
	T0 Rt	Tv Rt.	T1 Rt	T3 Rt	T0 Lt	Tv Lt.	T1 Lt	T3 Lt	T0 Rt	Tv Rt.	T1 Rt	T3 Rt	T0 Lt	Tv Lt.	T1 Lt	T3 Lt
pt01	3.9	3.9	3.4	4	1.8	1.7	4.1	2.5	3	3.2	4	3.1	3.2	3.3	4.3	3.3
pt02	2.3	2.3	2.8	2.1	1.7	1.6	2.6	2	3	3	3.7	3.3	3.7	3.5	3.3	3.3
pt03	1	1.1	2	1.2	1.3	1.3	2.1	1.5	2	1.9	3.1	2	1.5	1.5	2.3	1.8
pt04	2.4	2.3	2.5	2.5	2.3	2.2	2.2	2.4	4.8	4.1	5.7	4.6	4.3	4.1	3.6	4.7
pt05	2.3	2.4	2	2.1	1.6	1.5	2.1	1.4	2.1	2.5	2.2	2	2.9	2.6	3.5	2.9
pt06	1.8	1.6	3.5	2	1.7	1.9	1.9	2.2	1.2	1.2	2.9	1.2	1.2	1.1	2.1	1.4
pt07	3	3.1	4.2	3.3	2.9	2.5	3.5	2.6	2.3	2.2	4.5	2.5	2.8	2.4	3.8	2.6
pt08	2	2	2.5	1.9	1.4	1.4	3.3	1.8	2.6	2.6	2.6	2.6	2.4	2.4	3.8	2.2
pt09	1.1	1.2	1.9	1.9	1.1	0.8	2.5	2.5	1.8	1.8	2.9	1.6	1.6	0.8	3	1.8
pt10	2.1	1.8	3.8	2.3	1.3	1.6	2.8	1.3	2.4	1.9	4.4	2.8	1.5	2	3.9	2.1
pt11	1.4	1.4	3.7	1.6	1.3	1.3	1.4	1.3	1.5	1.5	3.9	2.7	1.5	1.5	2	1.7
pt12	3.3	3.3	3.6	3.2	2.2	2.6	3.6	3	3.1	3.1	2.7	3.1	3.4	1.9	3.6	3
pt13	1.3	1.2	1.2	1.1	1.1	1.2	2.8	1.6	1	1.1	3	1.3	2.3	2.3	4.8	2.3
pt14	1	1.7	1.3	1.5	1.2	1.3	1.7	1.4	1	1	1.4	1.2	1.1	1.6	1.6	1.3
pt15	1.4	1.5	2.4	1.6	1.6	1.2	1.8	1.8	3.1	2.2	4.4	2.7	1.7	1.4	4.2	2.4
pt16	2	2	2.8	1.7	3.5	3.4	3.2	2.6	1.8	1.8	3.4	1.8	4.8	2.4	7.5	3.1
pt17	2	2.2	2.1	2.6	2.4	2.2	2.1	2	2.4	1.9	2.6	2.8	5	3.2	3.4	4.5

pt18	2.2	1.4	1.6	2	1.8	2	3.2	2.6	3	1.8	3.3	2.5	2	3.8	4	3.2
pt19	1.7	2.6	2.1	1.9	1.7	1.1	2.8	1.3	2.5	2.5	4.3	1.6	2	1.4	3.8	1.7
pt20	2.8	2.3	3	2.6	1.8	1.4	2	1.8	3.8	3.4	3.7	3.6	2	2	2.1	2.1

	PJS								Angle(axial)							
	T0 Rt	Tv Rt.	T1 Rt	T3 Rt.	T0 Lt	Tv Lt.	T1 Lt	T3 Lt.	T0 Rt.	Tv Rt.	T1 Rt.	T3 Rt.	T0 Lt.	Tv Lt.	T1 Lt.	T3. Lt.
pt01	2.8	2.7	1.5	2.6	2	1.8	1.5	1.6	74.5	72	72.4	74	72.8	65.1	66.3	69.3
pt02	2.8	2.7	2.2	2.3	4.4	4.2	3	3.7	73.6	70.1	66.9	73.3	76.2	71.6	70.5	75.7
pt03	1.3	1.4	2.2	1.5	1.6	1.6	1.9	1.6	76.7	72.8	71	75	83	78.4	76.2	82
pt04	2.9	2.8	3.6	3	3.9	2.9	2.8	3.1	69.1	70.2	68.9	65	73.3	71.5	69.9	66.4
pt05	2.6	2.7	1	2.1	2.7	2.7	3.8	2.6	73.4	72	74.6	67.1	78.4	73.9	71.6	70.6
pt06	1.3	1.2	2.5	1.2	1.2	1	1.1	1	68.2	65.7	65.4	67.5	65.8	61.9	61.7	64
pt07	1.7	1.5	2.2	1.7	1.7	1.3	2.4	1.8	66	62	61.2	76.5	62.9	59.1	58.7	71.9
pt08	1.7	1.7	1.9	2.1	1.6	1.6	2.1	2	71.1	70	70.1	65	71.2	68	69.7	68
pt09	1.2	1.3	2.4	1.5	1	1.4	1.8	1.6	67.9	63	63.6	74.1	70.6	69	66.4	75.9
pt10	2.3	1.9	3.9	2.6	2.6	2.3	3.2	2.8	71.4	66	64.3	69.5	73.7	70	69.1	70.2
pt11	0.9	0.9	3	2.5	1.2	1.2	2.3	1.3	59.3	57.5	57.4	58	69.8	67.5	67.6	67.5
pt12	2.4	2.4	1.8	2	1.7	1.5	2.6	2	80.6	80.6	77.3	80	77.5	82	75.2	79.2
pt13	1.1	1.3	3.6	1.2	2.1	2.4	5.3	2.2	61.6	57.8	55.6	77.2	67.6	71	65.1	78.8
pt14	0.7	0.6	1.4	0.8	1.3	1.3	1.5	1.4	88.6	92.3	92.5	89	81.7	75	73.2	80.2
pt15	2.4	2.1	3.9	2.6	1.9	2.4	4.2	2.9	68.6	72.2	68.3	70.2	61.2	56.5	56.2	64.1
pt16	1.5	1.5	2.1	1.7	2.9	2.2	4.3	2.1	72.1	70	71.4	71	56.7	51.5	53.8	58.5

pt17	1.5	1.8	1.4	1.3	4.7	5.2	4	3.2	78.2	75	76.2	77.5	74.9	71	69.6	71.9
pt18	2.8	1.9	3.7	2.5	2.1	2.5	2.6	2.3	70.6	65.8	64.7	68.5	76.8	74	71.2	75.5
pt19	1.4	1.4	3.5	1.8	1.4	1.7	2.8	1.6	61.5	57	52.7	72.6	71.3	70	65.2	72.6
pt20	3.4	3.4	3.4	3.3	2.1	3.1	2.5	2.5	74.1	72	72.7	73.8	73	70	72.8	72

Supplementary Table S2 Raw data for oral functional evaluation

	MMO (mm)		lateral movement (right side) (mm)		lateral movement (left side) (mm)	
	preoperative	1 year postoperative	preoperative	1 year postoperative	preoperative	1 year postoperative
Pt01	49	48	10	12	11	10
Pt02	50	49	11	10	10	11
Pt03	48	50	12	11	12	11
Pt04	52	54	11	11	11	12
Pt05	57	53	12	13	13	12
Pt06	48	49	11	12	10	12
Pt07	53	53	14	12	13	13
Pt08	46	49	12	10	10	11
Pt09	52	54	13	14	12	12
Pt10	57	54	11	12	15	13
Pt11	56	55	14	12	13	14
Pt12	57	59	15	13	12	14
Pt13	58	55	14	15	15	14

Pt14	48	51	15	15	14	14
Pt15	55	53	12	13	12	13
Pt16	57	58	15	14	13	12
Pt17	58	57	11	12	12	12
Pt18	56	59	12	13	14	13
Pt19	52	53	14	14	12	12
Pt20	59	56	13	14	12	12
